# Critical Transitions in Early Embryonic Aortic Arch Patterning and Hemodynamics

**DOI:** 10.1371/journal.pone.0060271

**Published:** 2013-03-21

**Authors:** William J. Kowalski, Onur Dur, Yajuan Wang, Michael J. Patrick, Joseph P. Tinney, Bradley B. Keller, Kerem Pekkan

**Affiliations:** 1 Department of Biomedical Engineering, Carnegie Mellon University, Pittsburgh, Pennsylvania, United States of America; 2 Molecular Biosensor and Imaging Center, Carnegie Mellon University, Pittsburgh, Pennsylvania, United States of America; 3 Department of Pediatrics, Cardiovascular Innovation Institute, University of Louisville, Louisville, Kentucky, United States of America; New York Medical College, United States of America

## Abstract

Transformation from the bilaterally symmetric embryonic aortic arches to the mature great vessels is a complex morphogenetic process, requiring both vasculogenic and angiogenic mechanisms. Early aortic arch development occurs simultaneously with rapid changes in pulsatile blood flow, ventricular function, and downstream impedance in both invertebrate and vertebrate species. These dynamic biomechanical environmental landscapes provide critical epigenetic cues for vascular growth and remodeling. In our previous work, we examined hemodynamic loading and aortic arch growth in the chick embryo at Hamburger-Hamilton stages 18 and 24. We provided the first quantitative correlation between wall shear stress (WSS) and aortic arch diameter in the developing embryo, and observed that these two stages contained different aortic arch patterns with no inter-embryo variation. In the present study, we investigate these biomechanical events in the intermediate stage 21 to determine insights into this critical transition. We performed fluorescent dye microinjections to identify aortic arch patterns and measured diameters using both injection recordings and high-resolution optical coherence tomography. Flow and WSS were quantified with 3D computational fluid dynamics (CFD). Dye injections revealed that the transition in aortic arch pattern is not a uniform process and multiple configurations were documented at stage 21. CFD analysis showed that WSS is substantially elevated compared to both the previous (stage 18) and subsequent (stage 24) developmental time-points. These results demonstrate that acute increases in WSS are followed by a period of vascular remodeling to restore normative hemodynamic loading. Fluctuations in blood flow are one possible mechanism that impacts the timing of events such as aortic arch regression and generation, leading to the variable configurations at stage 21. Aortic arch variations noted during normal rapid vascular remodeling at stage 21 identify a temporal window of increased vulnerability to aberrant aortic arch morphogenesis with the potential for profound effects on subsequent cardiovascular morphogenesis.

## Introduction

Congenital heart disease (CHD) has the highest incidence and mortality rate of all birth defects in the U.S., occurring in at least 8 of every 1000 live births, and accounting for more than 24% of birth defect related infant deaths [1]. Due to the prevalence and severity of CHD, the cardiovascular (CV) system has become one of the most widely researched areas in developmental biology. Compared to other organ systems, the CV system is the first to form and the only one which is required to function successfully for survival [2]. Much of what we know about vertebrate CV development originated using the chick embryo, which undergoes cardiogenesis similar to humans and is amenable to both acute and chronic imaging and instrumentation [3]. Appearing at Hamburger-Hamilton stage 10 (33 h) in the chick, the heart is initially an open ended tube located at the ventral midline of the embryo and aligned parallel to the cranio-caudal axis [4], [5]. Cardiac looping transforms the heart into a looped tube by stage 24 (4 days) [6] and subsequent septation events produce the four-chambered heart and two great arteries, with all major CV structures formed by stage 36 (10 days) [7].

**Figure 1:**
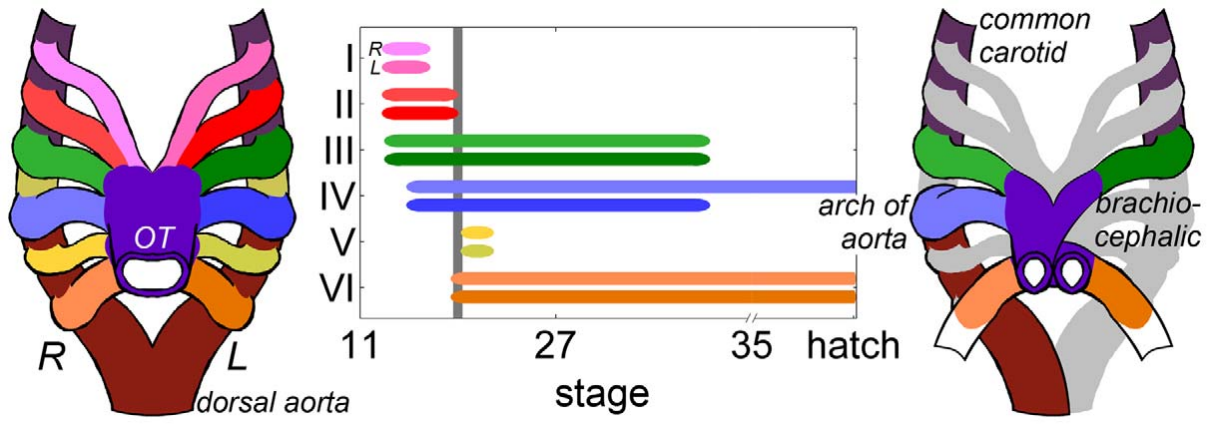
Timeline and schematic of the AA derivatives in the chick embryo. The timeline depicts the duration of the connection between the heart and descending dorsal aorta, where line width is an approximation of the frequency that the AA is present. The time axis is skewed to highlight the early stages investigated in this study. Though AA V is included here, it never truly connects to the dorsal aorta. The gray line designates stage 21, where up to four AA can be present. The schematic on the right depicts the mature avian great vessel pattern, where gray represents embryonic AA sections that disappear prior to the final arch configuration.

Unlike the adult circulation, where the right and left ventricles eject through semilunar valves into the pulmonary and systemic arterial circulations, respectively, the embryonic ventricle ejects blood through multiple, bilaterally paired aortic arches (AA). In the chick embryo, a total of six AA pairs (numbered I-VI) emerge consecutively in a cranio-caudal fashion, with three pairs generally co-existing at early embryonic time-points (Figure 1). This network of parallel vessels is selectively reduced and remodeled into the mature asymmetric aortic arch and pulmonary arteries by stage 36 (10 days) [8], [9]. Only three of the six AA pairs persist (III, IV, VI). Cranial-most AA I and II remodel into capillary beds and AA V exists only as a transient segment of AA VI [8]. AA III forms portions of the brachiocephalic and common carotid arteries. The right lateral AA IV forms a segment of the transverse adult aortic arch while the left lateral AA IV regresses. This asymmetric AA IV pattern is different in mammals: the left lateral AA IV contributes to the adult aortic arch and the right lateral AA IV forms a short segment of the proximal right subclavian artery. The caudal-most AA VI contributes to segments of the central pulmonary arteries and ductus arteriosus. This sequence of growth and remodeling events is vulnerable to genetic and epigenetic insults, and errors in AA morphogenesis occur in more than 20% of all CHD [1].

The AA transformation patterns detailed above were initially described through India ink injection and serial section experiments in the chick embryo [10], [11], [12], [13]. The first complete 3D analysis of AA morphogenesis used corrosion casts and scanning electron microscopy to generate detailed morphology from chick embryo stage 12 to hatching, providing a contemporary timeline of AA development (Figure 1) [8]. Advances in imaging technology, including micro-computed tomography (micro-CT) [14], magnetic resonance microscopy (MRM) [15], and optical coherence tomography (OCT) [16] now support comprehensive high-resolution studies of the 3D morphology of chick embryonic vasculature. Despite these advances, work related to AA morphogenesis has been predominantly descriptive and a lack of morphometric data persists. Studies that report measures of AA diameter often apply dehydration and fixation methods prior to acquiring measurements, which can distort vascular geometry [12], [17].

Multiple studies on the relationship between blood flow and vessel geometry have demonstrated what is referred to clinically as the “flow-dependency principle” [18], [19], [20], [21], establishing hemodynamics as a major epigenetic factor in vascular growth and remodeling. Wall shear stress (WSS), which is sensed by the endothelial cells, functions as a major extrinsic mechanical stimulus for vascular remodeling [22], [23], and prolonged exposure to altered blood flow results in the normalization of WSS via an increase or decrease in vessel caliber [18], [24], [25]. The role of hemodynamics in regulating vascular growth has been validated in a variety of species, including the zebrafish [26], [27], [28] and chick [29], [30], [31], [32], [33], [34], [35]. This relationship has been demonstrated for the global (organ scale) growth of the AA, where epigenetic perturbations in blood flow lead to congenital defects affecting the great vessels [35], [36], [37], [38].

While these studies provide evidence for the role of hemodynamics in AA growth and remodeling, limited quantitative spatial and temporal data on AA morphometry and flow have been available for biologists and bioengineers. In our previous work [39], we quantified changes in AA geometry, blood flow, and WSS between stages 18 and 24 in the chick embryo (3 and 4 days, respectively). Our *in vivo* measurements demonstrated that both AA III reduce in diameter while both AA IV increase in diameter. Using composite three dimensional (3D) AA models reconstructed from micro-CT scanning, we conducted stage-specific computational fluid dynamics (CFD) simulations to quantify AA blood flow and spatial variations in WSS. The results revealed a significant shift in the distribution of cardiac output to the individual AA; in particular, the AA that received the largest amount of flow changed from AA III at stage 18 to AA IV at stage 24. WSS values in the AA increased from stage 18 to 24, with the largest increase occurring in AA IV. This change in WSS was correlated with the enlargement of AA IV diameter, providing the first quantitative evidence for flow-dependent growth in the embryonic AA.

Our previous study also demonstrated that during the 24 hour period between stages 18 and 24, cranial AA II degenerates to a capillary bed and the caudal-most AA VI emerges. AA II, III, and IV were present in all stage 18 embryos while AA III, IV, and VI were present in all stage 24 embryos. While this stage-specific lack of inter-embryo variation in AA configuration seems to indicate a controlled developmental process, there have been no investigations of the intermediate stages to determine how this transition occurs. Should the intermediate stages present with uniform AA configurations, then it is possible that this transition is a tightly controlled and prescribed developmental program regulated by inherent temporal genetic activation. However, significant intra-stage variations in AA architectures would support the alternate hypothesis that epigenetic and environmental factors such as fluctuations in AA hemodynamics could be involved in determining final AA fates.

In the current study, we investigated the transitional stage 21 (3.5 days), applying the multimodal 3D quantitative approach established in our previous work. *In vivo* imaging of stage 21 AA was performed using fluorescent dye microinjections and OCT to acquire quantitative structural data without disrupting the morphology of the embryo. Representative 3D models of the stage 21 AA were reconstructed from micro-CT scans and used for CFD analysis. We demonstrate that multiple AA configurations exist in “normal” stage 21 embryos, suggesting a possible vulnerable window in the transition in AA configuration from stage18 to stage 24. CFD analyses indicate that variations in cardiac output distribution may be a critical factor in AA growth and selection by disrupting the timing of events such as AA regression, generation, and asymmetric growth. Thus, defining critical windows of developmental plasticity and the role of epigenetic, environmental factors that impact these developmental trajectories will help identify the origins of CV malformations and provide insights into the optimal timing for fetal intervention strategies to restore normal biomechanical loading, growth, and adaptation. [40].

## Methods

### *In vivo* aortic arch diameter measurement

**Figure 2:**
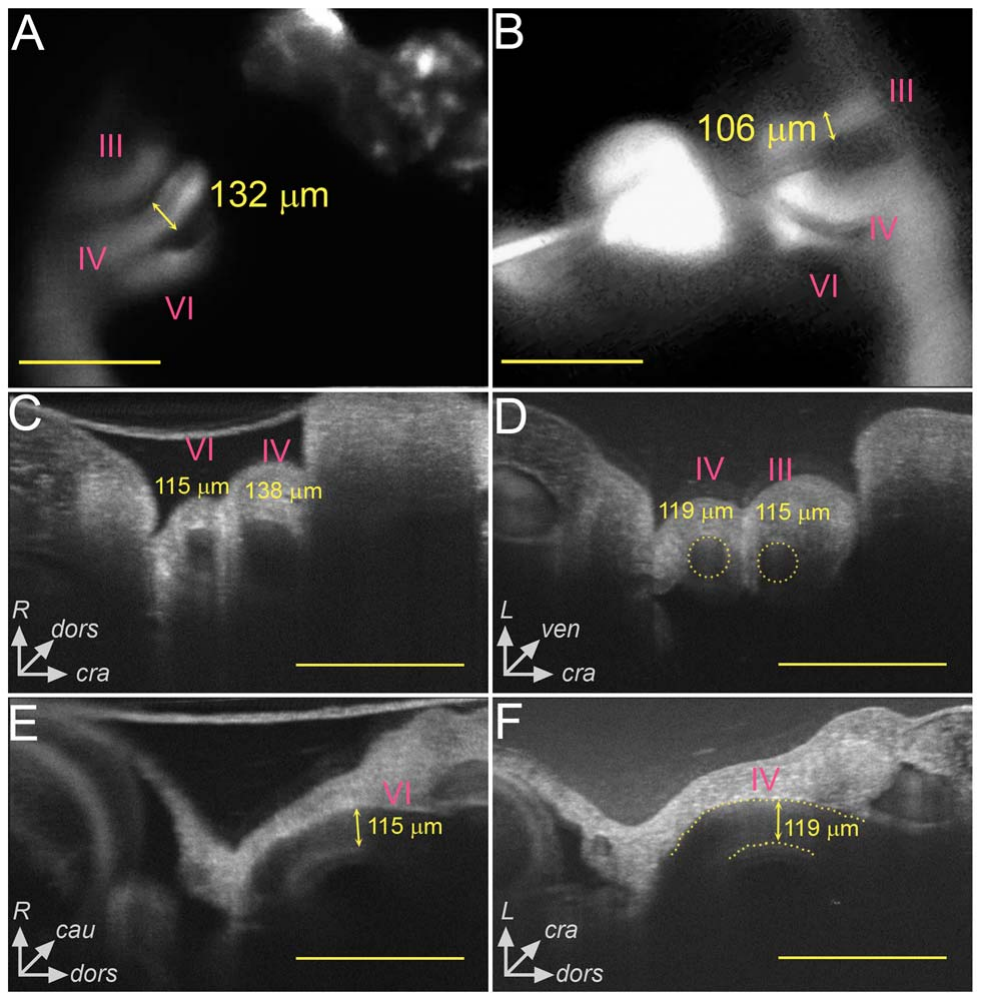
Multimodal imaging was used to obtain *in vivo* AA diameter measurements in stage 21 chick embryos. Video recordings from fluorescent dye microinjections were processed to obtain individual AA identity and midpoint diameter data (A,B). OCT was used to obtain transverse (C,D) and longitudinal (E,F) sections through the AA to analyze midpoint diameter and AA tapering. Both the right (A,C,E) and left (B,D,F) laterals were imaged. In D, the dotted circles represent the AA lumen based on our image processing algorithm. The dotted lines in F highlight the boundaries of the AA lumen. Comparison between imaging modalities showed good agreement (p>0.05). The axes in C-F demonstrate the dorsal (dors), ventral (ven), cranial (cra), caudal (cau), right (R), and left (L) directions to orient the reader. All scale bars are 500 µm.

Fertilized white Leghorn chick eggs were incubated at 37°C and 60–70% relative humidity to stage 21 (3.5 days). We windowed the shell and removed the overlying membranes to expose the embryo and gain optical access. Using our fluid microinjection technique, we injected embryos with approximately 0.5 μl of Rhodamine B diluted in PBS [41]. We recorded time-lapse movies of each injection and extracted still frames for further analysis. The left and right lateral AA were identified using anatomical landmarks and AA midpoint diameters were then measured (Figure 2). A total of three measurements were made on each vessel and then averaged to obtain the midpoint diameter. AA identity and diameter measurements were performed by three independent observers, and inter-observer agreement was assessed using Bland-Altman analysis [42]. A total of 32 right-lateral and 18 left-lateral stage 21 embryos were injected and analyzed.

Dye injection data was confirmed using a spectral domain OCT (SDOCT) system (Thorlabs Spectral Domain Ganymede, Thorlabs, Inc., NJ) to acquire noninvasive, *in vivo* images of the right and left lateral AA (4.3 μm resolution). OCT is an echo-based modality, which uses low-coherence interferometry to measure the axial distance of back-reflected light [43]. We have previously applied and validated our OCT system in the development of a novel velocimetry technique for live embryos [44]. The OCT light source is comprised of a 930 nm center wavelength (*λ*) superluminescent diode with a spectral bandwidth (Δ*λ*, FWHM) of 100 nm. The 930 nm source is within the 600–1300 nm “therapeutic window” for optical radiation, while the total optical power on the sample was 1.5 mW, producing no thermal damage [45], [46], [47]. Sensitivity of the OCT system defines the minimum detectable change in index of refraction and was measured experimentally to be 91 dB (manufacturer specification). The theoretical axial resolution depends on the coherence length of the OCT light source, and is expressed as 2ln(2)*λ* ^2^/Δ*λπn*, where *n* is the refractive index of the sample medium [48]. In SDOCT, the design of the spectrometer and signal processing both affect the actual resolution. The spectrometer used in our OCT system can image a spectral range (*δ*) of 150 μm, while the OCT software applied Hann-windowing of the spectrum to give smooth axial point-spread functions. The actual axial resolution of our system was *λ* ^2^/(*δn*), equivalent to 5.8 μm in air and 4.3 μm in water. Lateral resolution is set by the minimum waist radius of the focused OCT beam, which in our system was 15 μm. The spectrometer used in our OCT system consisted of a 12 bit high-sensitivity CCD camera with 2.0 μm pixel spacing. Data was transferred in real-time over a GigE connection to a PC with a 3.3 GHz processor. The maximum A-scan rate of our OCT system was 29 kHz (equivalent to 38.3 fps for 757 A-lines per frame). The rate of data transfer and live streaming of the 2D OCT scan produced an actual recorded frame rate of 17.1 fps for a 757 A-line image (12.9 kHz). The sample refractive index is defined for the medium surrounding the sample and was considered 1.33 for *in ovo* embryo imaging.

Eggs were windowed as described above and placed in a temperature and humidity controlled imaging chamber. We acquired time-resolved 757×757 (1.5×1.5 mm) 2D transverse image sequences, each lasting approximately five cardiac cycles. As the AA curves around the foregut to connect to the dorsal aorta, its proximal portion has a significant lateral orientation while the mid to distal region is oriented predominantly dorso-ventral. We acquired transverse sections approximately halfway between the start of the dorso-ventral orientation and the connection to the dorsal aorta (Figure 2). Sequential 2D images were averaged in order to identify the AA lumen by negative contrast (Figure 2). Red blood cells produce a transient reflection as they pass through the scanning beam, and applying an intensity-average removes these areas while maintaining the constant signal from the surrounding tissue. We applied an *ad hoc* image processing code to quantitatively measure AA diameter from OCT scans. Approximately 10 discrete points marking the boundary of the AA lumen were manually selected from an intensity-averaged 2D transverse image. The centroid and radius were then computed by fitting the circle equation to the selected points. A total of 17 right lateral and 10 left lateral embryos were analyzed using OCT, with one measurement per AA per embryo. We performed two-tailed, unpaired t-tests assuming equal variance to determine significant differences (p<0.05) in AA diameters at stage 21 (right vs. left lateral of the same AA pair, right laterals compared against each other, left laterals compared against each other).

For some of the stage 21 embryos, the full AA lumen could not be visualized with OCT due to excessive light scattering at the air-egg interface and through the pharyngeal arch tissue (Figure 2). During the manual identification of the lumen, we only selected points where the boundary was clearly visible, normally encompassing an arc length of 1/2 to 2/3 of the total circumference. To test whether this truncated arc affected our measurements, we applied our technique to a phantom vessel of known diameter. Our phantom consisted of a clear nylon fiber (Stren Original 4 lb monofilament fishing line, Pure Fishing, Inc., SC) submerged in water. The expected fiber diameter, measured with a micrometer, was 203 μm. We acquired a 757×757 transverse image of the phantom using our OCT system and selected 14 points marking the fiber boundary, including 9 points distributed along the top 1/2 of the circumference and 5 points along the bottom 1/3. We then computed four diameter measurements, each using different subsets of the total 14 points selected to determine if the circular arc length contained by the selected boundary affected our measurement (Figure S1). The OCT measured fiber diameter was 240 μm and only varied by 2 μm when the number and circumferential distribution of the selected points changed. This test demonstrated that the truncated arc selected during AA measurements will produce a valid result and that the entire cross section does not need to be visible. The OCT measured diameter was larger than the expected fiber diameter (18% error), which may indicate over-estimation when applying our measurement technique to the AA. Considering the average AA diameter of 113 μm (Table 1), this error indicates our measurements are accurate to within 20 μm and is within the standard deviation (SD) of the AA diameters. However, the discrepancy in the fiber measurement may also be due to the tolerance of the micrometer.

**Table 1:** Average AA midpoint diameter (±SD) for all four possible AA present at stage 21.

AA	Midpoint diameter (±SD) (mm)			
	R		L	
II	0.109	(0.009)	0.110	(0.024)
	n =	9	n =	8
III	0.125	(0.020)	0.114	(0.019)*
	n =	39	n =	27
IV	0.123	(0.021)	0.115	(0.021)^†^
	n =	49	n =	28
VI	0.118	(0.026)	0.094	(0.027)*,†
	n =	41	n =	14

* ,† indicate a statistically significant difference (p<0.05) between AA diameters.

To further assess the diameters computed from the circle fitting method, we acquired longitudinal AA sections for those embryos where the inner-most wall of the AA was visible under OCT (Figure 2, note that the inner-most wall is towards the bottom). A total of four longitudinal AA images (2 right, 2 left) were acquired for this comparison, and we obtained three diameter measurements per AA at different points along the vessel length. The measurement from the longitudinal section taken at approximately the same position as the transverse section agreed well with the diameter computed from the circle fitting method in all cases (within ±5 μm, we did not perform statistical t-tests due to the insufficient sample sizes). The diameters reported in this manuscript only refer to those from the transverse images, as the longitudinal sections were only used to check the fairness of those measurements. The longitudinal measurements, and that the OCT and fluorescent dye measurements agree, suggest that our OCT data are an accurate measurement of AA lumen diameters.

### 3D aortic arch imaging and reconstruction

We injected a rapidly polymerizing resin (diluted MICROFIL® Silicone Rubber Injection Compounds MV-blue, Flow Tech Inc, Carver, MA) into stage 21 embryos to obtain 3D casts of the AA, as previously described by our group [39]. Casts were scanned using micro-CT (Scanco Inc.) and we reconstructed 3D models using our established protocols [39]. We acquired micro-CT scans of 20 embryos. Several selected scans were imported into computer-aided modeling software (Geomagics Inc., Durham, NC) and combined to create a representative AA geometry with smooth inflow/outflow boundaries required for CFD. This baseline model contained AA III and IV, as they are the two vessels present across stage 21 (see Results below). Using fluorescent injection and OCT imaging as a guide, we transformed the baseline configuration into the other three patterns observed at stage 21 by adding AA vessels using our sketch-based 3D anatomical editing tool [49]. The 3D models compared well with experimental measurements (Tables S1 and S2), supporting realistic data from CFD analysis.

### Computational fluid dynamics simulation and analysis

We performed 3D CFD simulations as previously described [39]. A pulsatile 2^nd^-order CFD solver (Fluent 6.3.26, ANSYS Inc.) simulated blood flow through the AA models, applying rigid, no-slip walls and Newtonian assumptions (ρ = 1060 kg/m^3^, μ  = 3.71×10^−3^ Pa–s) [50]. We prescribed time-dependent flow waveforms as plug-flow inflow boundary conditions, based on our previously published outflow tract velocity measurements (Figure S2) [51]. The distribution of cardiac output to the trunk and cranial vessels was set at a ratio of 90/10 using flow-split boundary conditions [52]. Steady-state solutions were used to initialize the flow field prior to transient solutions. Convergence was enforced by reducing the residual of continuity equation by 10^−6^ at all time steps. Flow variables were monitored in real-time at the aortic inlet and descending aorta outlet during the course of each solution to ensure that nonlinear start-up effects were eliminated. A mesh sensitivity study at three refinement levels was performed to assure grid independency. Six cardiac cycles were simulated and required approximately 48 h on a Linux workstation with two Quad Core Intel Xeon processors (8 nodes each 2.66 GHz) with 8GB of shared parallel memory.

## Results

### Variation of aortic arch number and type at stage 21

**Figure 3:**
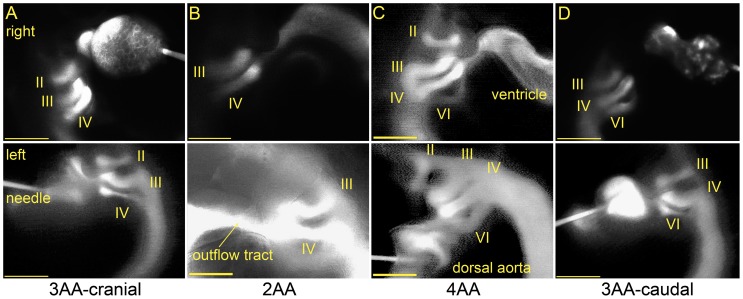
Four distinct AA configurations were observed at stage 21, identified through fluorescent dye microinjection. Right (top) and left (bottom) lateral snapshots from video recordings are shown. (A) The 3AA-cranial pattern includes AA II, III, and IV. (B) The 2AA pattern only includes AA III and IV. (C) AA II, III, IV, and VI are present in the 4AA configuration. (D) The 3AA-caudal pattern includes the caudal-most AA VI. All scale bars are 500 µm.

In our previous study of stage 18 and 24 embryos, we observed no inter-embryo variation in AA configurations; stage 18 contained AA II, III, and IV while stage 24 contained AA III, IV, and VI [39]. In the present investigation of stage 21 embryos, however, we identified four different AA configurations based on visual inspections of fluorescent dye injections, demonstrating significant variability (Figure 3). Anatomical landmarks, such as pharyngeal arch 2, were used to identify the AA present. Two three AA patterns were observed, which we refer to as 3AA-cranial (AA II, III and IV present) and 3AA-caudal (AA III, IV, and VI present). A two AA configuration, 2AA, was found with only AA III and IV present. A four AA configuration, 4AA, displayed AA II, III, IV, and VI. The 3AA-cranial contains the same AA as stage 18 while the 3AA-caudal includes the same AA as stage 24 [39]. The 2AA and 4AA patterns are unique to stage 21. All four configurations were observed for both laterals in at least two embryos. The 3AA-caudal configuration appeared the most frequently (n = 20 right, n = 8 left), followed by 4AA (n = 5 right, n = 5 left), 3AA-cranial (n = 4 right, n = 3 left), and finally 2AA (n = 3 right, n = 2 left). Although lacking simultaneous left and right lateral measurements in the same embryo, our analysis confirmed bilateral symmetry of all AA configurations by observing the flipped embryos after injection and cessation of heart beat.

We did not observe AA V in any of our fluorescent dye injections. Polymeric casts suggest that AA V branches from and then reconnects to AA VI prior to anastomosis with the dorsal aorta and that it is significantly smaller than the other AA. [8]. The large size of pharyngeal arch 2 allowed us to identify which AA vessels were present with little difficulty and there were no disagreements when comparing independent observer classifications. AA V may not be fully formed by stage 21 or does not receive significant flow to produce a fluorescent signal; therefore, we cannot confirm the presence of AA V at stage 21.

### Dimensions of the stage 21 aortic arches

**Figure 4:**
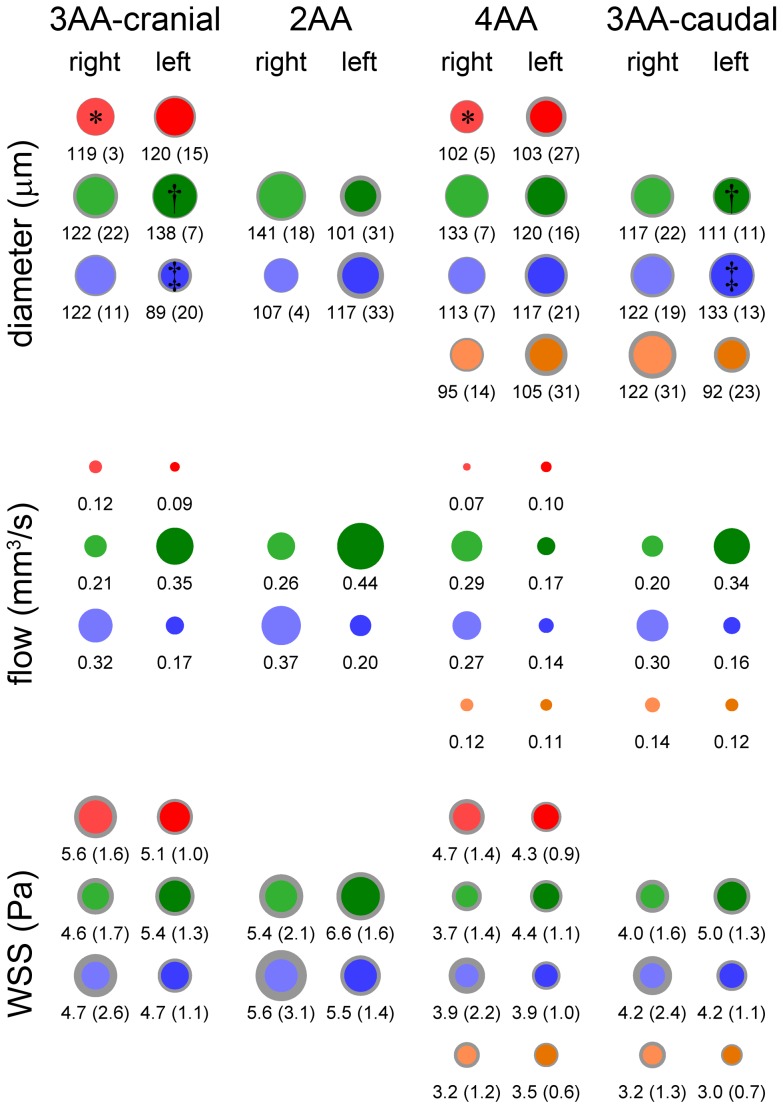
Average (±SD) AA midpoint diameters, cardiac cycle-averaged flows, and spatially-averaged (±SD) cycle-average WSS levels for each of the four configurations present at stage 21. AA diameters are scaled, with values given below. Gray boundaries give the SD. *,†,‡ indicate significant differences (p<0.05) in AA diameter.

Average mid-point AA diameters were measured from fluorescent dye injections after classification of the AA configuration. The inter-observer bias in fluorescent dye measurements was 2 μm and the limits of agreement were −26 to 29 μm, demonstrating reasonable agreement. AA measured with OCT were not classified, and data from OCT was combined with all fluorescent dye data to generate the average stage 21 diameter measurements (Table 1). Fluorescent dye injection and OCT measurements were unmatched, though the average difference between mean diameters obtained using the two methods was 5 μm, suggesting close concordance. Further, a two-tailed, unpaired t-test did not show significant differences between the two methods (p>0.05). Statistical comparison of the stage 21 AA diameters revealed that the left lateral AA VI was smaller than the left lateral AA IV and III (p<0.05, Table 1). Using only the classified fluorescent dye data, we performed further analysis to determine if significant differences in AA diameter existed between the AA configurations. For the vast majority, no significant differences were found; however, the right lateral AA II was larger in the 3AA-cranial vs. 4AA configuration, and the left lateral AA III was larger and the left lateral AA IV smaller in the 3AA-cranial vs. 3AA-caudal configuration (Figure 4). These data indicate that although AA configurations vary at stage 21, AA diameters are fairly uniform throughout the stage 21 time period. However, we also recognize that the small sample size of certain AA configurations (i.e. 2AA) may limit statistical comparisons. We also compared the average stage 21 diameter data with our previous measurements at stages 18 and 24 [39] and found significant differences (p<0.05) between both AA IV laterals from stage 18 to 21, and between the right lateral AA IV and both AA VI laterals from stage 21 to 24 (Figure S3, Table S3). In all cases, the diameter was larger at the later stage.

### Distribution of cardiac output in the stage 21 aortic arches

Figure 4 depicts the cardiac cycle-averaged flow rates and spatially-averaged cycle-averaged WSS values calculated by CFD analysis for all four stage 21 configurations. Individual AA flow estimations from CFD agree well with previously published Doppler ultrasound flow measurements reported in the stage 24 chick embryo [36]. Flow through the AA manifold was laminar, with maximal Reynolds number of less than 20 at the junction between the outflow tract and aortic sac. Womersley numbers were less than 1 in all AA.

For all four configurations, AA III and IV were the most perfused, comprising 45% and 38% of the total cardiac output, respectively. AA II and VI received the least flow in the 3AA-cranial and 3AA-caudal cases, respectively, and were also the least perfused AA in the 4AA case (13% to AA II, 18% to AA VI of total cardiac output). Flow in the 2AA configuration was split almost evenly, with AA III receiving 55% of the total cardiac output compared to 45% for AA IV. For all but the 4AA configuration, cardiac output was split evenly between the right and left laterals; in the 4AA case, the right laterals received 60% of the total cardiac output. In the configurations in which they appear, AA II and AA VI received considerably less flow, though this disparity was less pronounced in the left laterals of the 4AA case. For the left laterals, AA III received the most flow in all configurations, though in the 4AA case flow was more evenly distributed among the left lateral AA. For the right laterals, AA IV received the greatest amount of flow for all but the 4AA configuration, in which AA III and AA IV were nearly equally perfused. Examining each AA pair individually, the right lateral of AA II received 57% of all AA II flow in the 3AA-cranial configuration, a distribution that was reversed in the 4AA case (left lateral AA II received 59% of AA II flow). AA III flow was consistently split 63% to 37%, between the right and left lateral, with the left lateral receiving the larger share for all but the 4AA configuration, in which the right lateral received 63% of the flow. In all cases, the right lateral AA IV received 65% of the cardiac output directed to AA IV. This was the largest difference (65% vs. 35%) between right and left lateral flow distribution for any AA pair. Flow to AA VI was split nearly evenly between its laterals, 52% right vs. 48% left for both configurations in which it appeared.

### Distribution of WSS patterns in the stage 21 aortic arches

**Figure 5:**
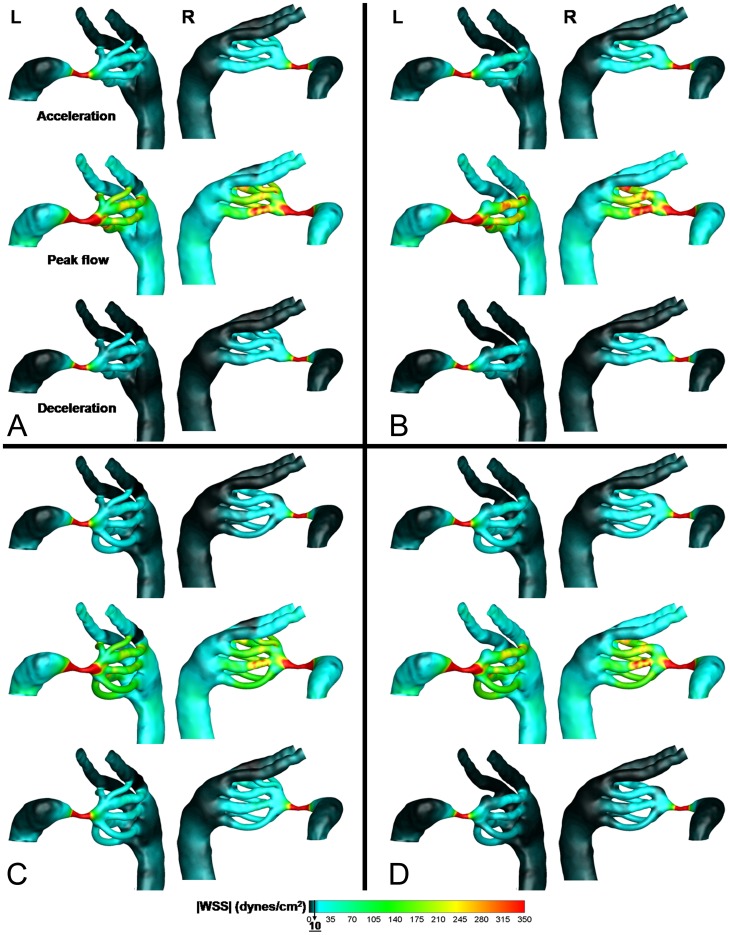
AA WSS distributions computed from CFD simulations for each of the four configurations observed at stage 21. Acceleration, peak, and deceleration phases of the cardiac cycle are depicted. (A) 3AA-cranial, (B) 2AA, (C) 4AA, (D) 3AA-caudal.

With respect to our previous data [39], WSS was elevated in all AA at stage 21 compared to the previous (stage 18) and later (stage 24) time-points (Figure S3). WSS levels at stages 18 and 24 were between 1 and 3 Pa, compared to the 3–7 Pa range at stage 21. Spatial distribution of WSS, including the acceleration, peak, and deceleration phases of the cardiac cycle, is depicted in Figure 5. The highest WSS zones were located at the junction between the outflow tract and aortic sac, and in the narrow segments of AA III. WSS levels were similar in all AA pairs, though AA VI levels were relatively lower in the configurations where it appears (Figure 4). Examining AA pair by pair, the WSS levels in the right lateral of AA II were always higher than its left lateral (0.5 Pa higher on average). This situation was reversed for AA III, where the left lateral was exposed to higher WSS levels (average of 0.9 Pa higher). As in the flow distribution, AA IV WSS levels were consistently higher in the right lateral, though this difference was less dramatic than the AA II and III WSS (less than 0.1 Pa on average). WSS levels in AA VI were also similar in both laterals, with an average difference of 0.3 Pa.

The increased WSS at stage 21 can induce significant changes in AA growth through shear-mediated genetic and signaling pathways. That WSS levels at stage 24 are similar to those at stage 18 suggests mechanical restoration, a theory introduced for biomechanically-regulated growth [53], [54]. This theory, in which tissues are expected to grow and remodel in an attempt to restore homeostatic or optimal loading conditions, has been demonstrated in limited embryonic applications [55], [56]. Additional research is required to determine target stress states in the embryo, which may change over the course of development.

### Correlation between WSS variation and diameter change

**Figure 6:**
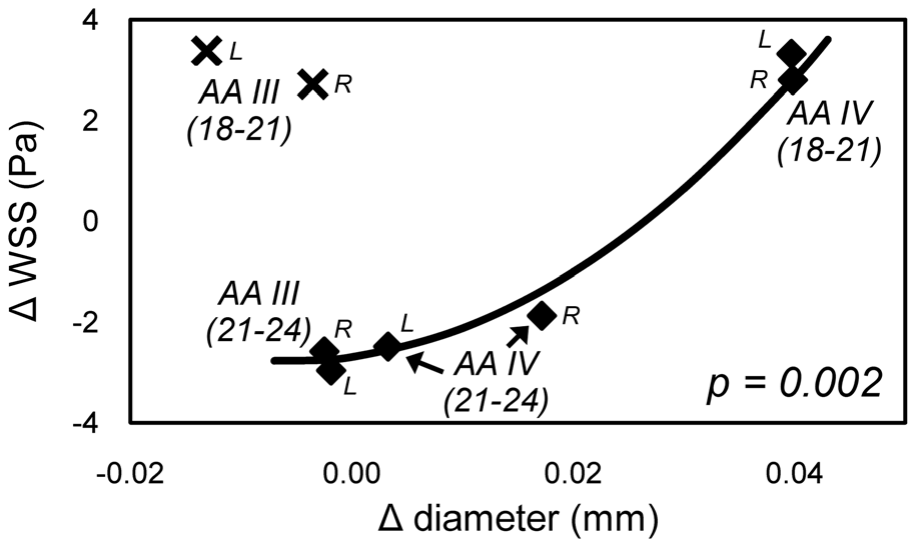
Comparing changes in diameter with changes in WSS from stages 18 to 21 and 21 to 24 for AA III and IV revealed a 2^nd^ order polynomial relationship (p = 0.002). Growth of AA IV from stage 18 to 21 and growth of both AA III and IV from stage 21 to 24 follow this trend.

In an attempt to further define the relationship between WSS and vascular growth, we determined if a correlation exists between an incremental change in vessel diameter and a change in WSS. We performed a regression analysis on the differences between average left and right lateral diameter and WSS values at stages 18 and 21 and stages 21 and 24 for AA III and IV. For six of the eight AA vessels, a 2^nd^-order polynomial function strongly correlated variation in WSS with change in diameter (p = 0.002, Figure 6), consistent with hyper-restoration theory. Outliers to this trend included both the right and left lateral of AA III during growth from stage 18 to 21. It is noteworthy that the change in WSS must exceed some threshold to produce a significant change in AA diameter, and increases in WSS have a greater effect than decreases. Deviations from this trend (i.e. WSS decreasing and diameter increasing) were observed when comparing each stage 21 configuration separately to the stage 18 and 24 data (Figure S3) and may be related to cellular heterogeneity within the AA, causing different responses to WSS levels.

## Discussion

### Inter-embryo variability in aortic arch patterns coincides with increased wall shear stress

**Figure 7:**
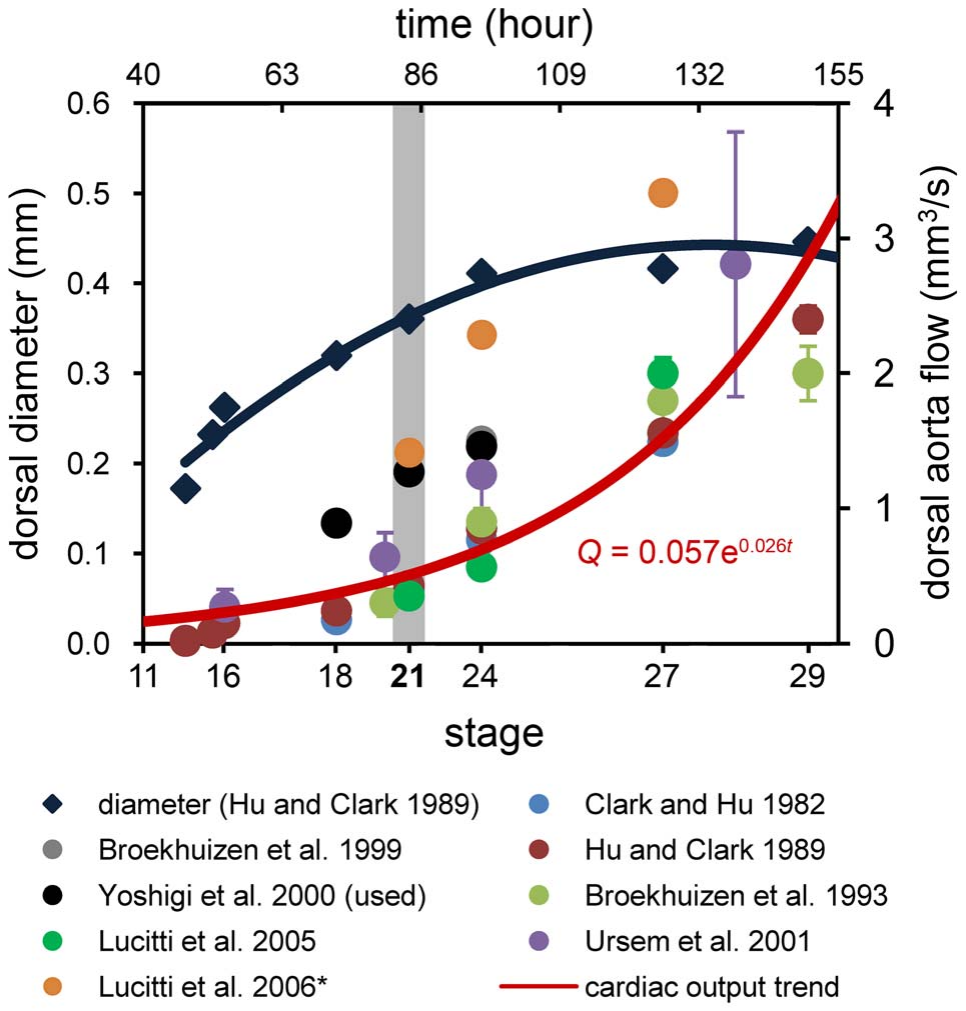
Cardiac output increases exponentially with developmental stage while dorsal aorta diameter lags. The rate of change can vary from embryo to embryo and may be related to the multiple AA configurations observed at stage 21 (highlighted in gray). * Lucitti et al. [60] reported velocity data only, which we converted to flow rate using dorsal aorta diameter data from Hu and Clark [52]. Equation of the exponential trend is given in the lower right corner, where *Q* is the flow rate and *t* is time, in hours.

Our fluorescent dye injections demonstrate significant variations in AA patterns at stage 21, which was not observed at previous (stage 18) or later (stage 24) developmental time-points. This inter-embryo variability was also noted by Pexieder, who documented similar observations at four hours prior to and after stage 21 [12]. Pexieder's observations underscored the importance of using developmental staging landmarks rather than duration of incubation time in assigning developmental stage to maturing avian embryos. CFD models of all four stage 21 AA configurations show a concomitant acute increase in WSS. Compared to our previous study of stage 18 AA [39], we found an average increase of 3.1 Pa (nearly 2 fold) per AA at stage 21; WSS then reduced 2.5 Pa (0.5 fold) by stage 24. This escalation in WSS is likely due, in part, to the exponential rise in cardiac output that occurs during development [31], [51], [52], [57], [58], [59], [60], [61], though more research is needed to determine these effects (Figure 7). Based on the coincidence between AA pattern variability and the transient sharp increase in WSS, we hypothesize that stage 21 represents a period of dynamic AA growth, regression, and generation events, which attempt to restore normal loading. While many experiments indicate the importance of flow distribution in AA growth and morphogenesis [35], [36], [38], the WSS after intervention remains unknown. Future work to characterize the biomechanical environment in these perturbed flow models is required to test this hypothesis.

### Asymmetric cardiac output distribution to the aortic arches

Our CFD results demonstrated clear differences in cardiac output distribution to AA pairs, as well as asymmetric perfusion between the laterals of distinct pairs (i.e. AA IV). Our group recently developed an optimization-based model for AA growth, where the individual AA diameters were free to alter in response to a global objective function that minimizes the total energy expenditure while maximizing diffusive capacity [62]. This model demonstrated that there was always one dominant (larger diameter) AA, the selection of which was strongly related to the orientation of the outflow tract. The outflow tract orientation acted to preferentially direct flow to one of the AA vessels, which became the dominant AA. This model showed similarities to the classic problem of competing collateral vessels, where small perturbations in the distribution of blood flow cause one vessel to dilate due to increased WSS while the others constrict due to a decrease in WSS, eventually leading to reduction to a single vessel [63], [64], [65]. Based on this work, the asymmetry observed in AA IV flow at stage 21 (right-lateral dominant, Figure 4) may explain the asymmetric growth of this AA pair, where the left lateral disappears and the right lateral forms a section of the mature arch of aorta. If we consider AA IV as two vessels competing for flow, then this flow asymmetry would predict degeneration of the left lateral. The low flow to AA II may also explain its eventual remodeling to a capillary bed by a similar principle. Asymmetric flow distribution was shown to affect platelet-derived growth factor-A and vascular endothelial growth factor receptor-2 signaling in the asymmetric remodeling of AA VI in the mouse, providing further support for this theory [66]. To date, inherent asymmetry in vascular growth-related gene expression among the AA has not been documented, suggesting that environmental, epigenetic factors such as WSS may play an important role in this process.

Multiple studies using intervention methods to disrupt normal flow in embryos near stage 21 have reported significant subsequent abnormalities in AA growth. Rychter and Lemez [38] tracked the distribution of blood from the vitelline veins in stage 13, 15, and 18 chick embryos, demonstrating clear patterns in AA perfusion. Exclusion of these veins by transection or ligation subsequently re-routed flow to AA not normally perfused from the tested location. Using India ink injections, Hogers et al. [35] extended this vitelline ligation model to demonstrate that intracardiac flow patterns were also disrupted. Further, embryos were examined through hatching, revealing multiple defects in AA development, including hypoplastic right brachiocephalic artery, interrupted aortic arch, double aortic arch, and hypoplastic pulmonary artery. Using only video microscopy, Hu et al. [36] reported similar anomalies in AA perfusion patterns in the left atrial ligated (LAL) chick embryo. Individual AA flow rates were quantified with laser Doppler velocimetry, and demonstrated a significant reduction of flow in all AA in the LAL embryos, although the flow ratios remained similar to the control group. Examination of LAL embryos to stage 27 and 34 revealed defects such as absent AA III and IV and AA hypoplasia.

It is possible to test the effects of altered outflow tract flow patterns using our current CFD models. As described in our CFD methods, the velocity profile at the outflow tract was plug shaped. The plug flow profile at the outlet of the beating ventricle is an established and valid assumption of cardiovascular fluid dynamics modeling [67], and is therefore employed in this study as well. To examine the effects of other profile shapes, we used the 3AA-cranial model and altered the inlet boundary condition. We prescribed two conditions: 1) a normal parabolic profile, with the maximum velocity occurring at the centroid of the inlet surface, and 2) a skewed parabolic profile, where the maximum velocity is offset from center. We modeled pulsatile flow, using the same stage 21 waveform (Figure S2), and kept the remaining boundary conditions and model parameters unchanged. Neither profile significantly altered the flow distribution, with an average difference of 1% (Table 2, Figure S4). This small effect is likely because the profile rapidly becomes fully developed (parabolic) by the time it reaches the aortic sac, even though we prescribe a plug-flow profile at the inlet. This flow development occurs particularly in the embryonic outflow tract since it has a narrow constriction upstream of our main area interest, the AA vessels. Furthermore, we expect that the narrowing of the outflow tract and the low Reynolds number attenuates (as a function of the constriction diameter) any flow skewness that may be exist due to the looping of the heart. These results seem to suggest that, even if the altered intracardiac flow patterns resulted in a skewed profile at the outflow tract, its effects on AA flow distribution would be small and possibly insufficient to cause morphogenetic abnormalities. However, the outflow tract of the early embryo through stage 32 is contractile and changes its shape during the cardiac cycle [68]. As our models do not incorporate this wall motion, the effects of the skewed profile may be underestimated. An AA flow model that incorporates the wall motion of the outflow tract would provide further evidence; however it is not within the scope of the current study.

**Table 2:** Stage 21 3AA-cranial flow (mm^3^/s) distributions using a plug, parabolic, and skewed parabolic inlet profile.

	plug		parabolic		skewed	
AA	R	L	R	L	R	L
II	0.120	0.090	0.121	0.089	0.121	0.089
III	0.210	0.350	0.210	0.351	0.209	0.351
IV	0.320	0.170	0.311	0.165	0.311	0.165

### Hypothetical model for transitions in aortic arch patterns at stage 21

**Figure 8:**
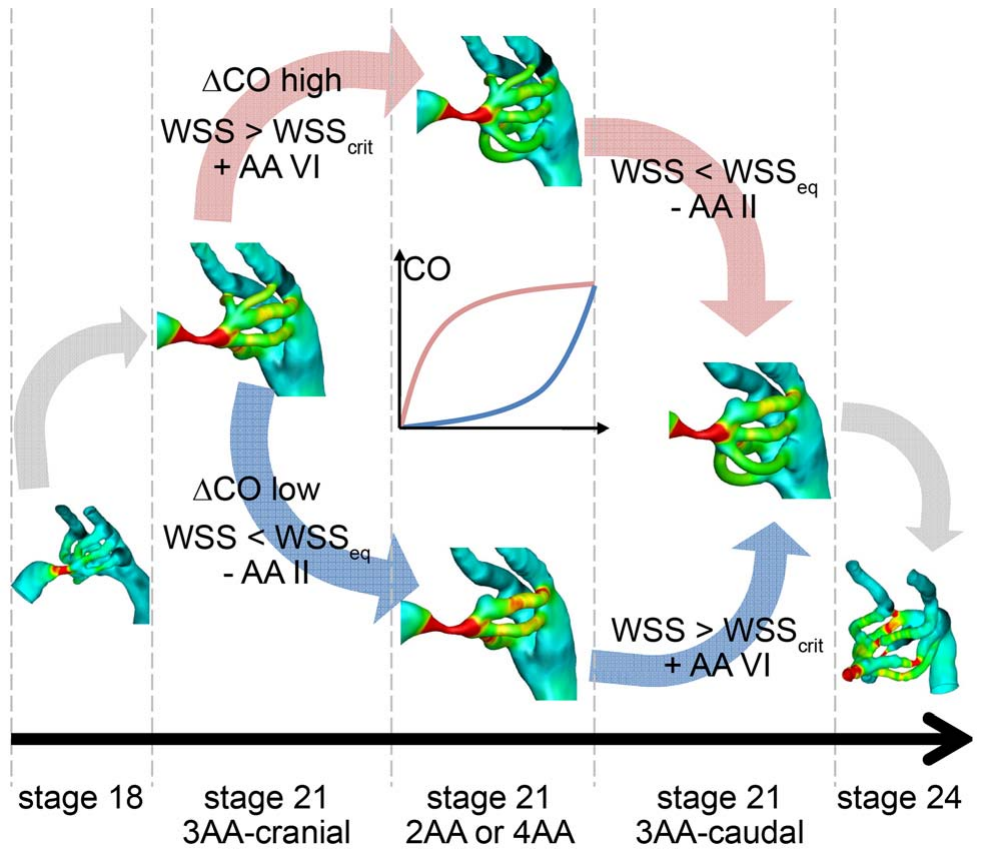
Theoretical growth pathways from stage 18 to stage 24 AA configurations. The transition can proceed through the 2AA or 4AA pattern depending on the timing of AA II regression and AA IV generation. WSS may be one factor in selecting the pathway, where levels above a critical value (WSS_crit_) lead to AA generation and WSS below an equilibrium level (WSS_eq_) leads to AA regression. The rate of increase in cardiac output (CO) can affect WSS levels.

The multiple AA configurations at stage 21 led us to propose two pathways by which the transition from the stage 18 to stage 24 AA patterns occurs (Figure 8). Each of the four AA configurations observed at stage 21 can represent a discrete snapshot occurring during the disappearance of AA II and emergence of AA VI. The 3AA-cranial configuration maintains the stage 18 II, III, IV AA, and can be considered as the immature stage 21 AA pattern. Similarly, as the 3AA-caudal configuration contains the same AA as the stage 24 embryo, it can be referred to as the mature stage 21. The remaining configurations, 2AA and 4AA represent two distinct intermediate stage 21 configurations, which, in turn, demonstrate the two possible growth pathways by which AA II degenerates and AA IV becomes patent. To achieve the 2AA configuration, AA II must degenerate before AA VI emerges; for the 4AA configuration to occur, AA VI must emerge before AA II disappears. While these two pathways can be logically deduced from our fluorescent injection data, the factors governing whether AA morphogenesis proceeds through the 2AA or 4AA pattern is unclear. Based on previous experiments demonstrating the importance of blood flow in AA growth, it is reasonable to propose that hemodynamic loading, such as WSS, has a role in this process. Mechanical restoration theory [53], [54] may offer some insight: prolonged exposure to WSS above some critical value (WSS_crit_) may lead to AA generation while WSS far below the normative level (WSS_eq_) may lead to AA regression in order to restore loading to WSS_eq_ (Figure 8). Variations in the trend of cardiac output increase is one possible explanation for the selection of either the 2AA or 4AA pathway, where a sharp increase would lead to generation of AA VI and a slow increase in cardiac output would lead to regression of AA II. Simultaneous hemodynamic and structural measurements, which are not currently available, are needed to investigate this theory.

### Relating wall shear stress to biologic events

Several published studies related to the biomechanical regulation of genetic, signaling, and cellular events involved in the normal growth and remodeling of the embryonic AA place the results from our CFD models into context with AA biology. Groenendijk et al. found that levels of high WSS were associated with Krüppel-like factor-2 (*KLF-2*) and endothelial nitric oxide synthase (*NOS-3*) expression, while low WSS areas expressed endothelin-1 (*ET-1*) [69]. Their 3D reconstructions of these expression patterns qualitatively overlap with the WSS magnitudes predicted by our current and previous CFD models [39]. Egorova et al. demonstrated that chick endothelial cells have a dose-dependent relationship between WSS and Tgfβ/Alk5 signaling activity [70]. Defective Alk5 signaling in mouse neural crest cells lead to AA hypoplasia and uncharacteristic regression [71]. The asymmetric WSS in AA pair IV may result in asymmetric Alk5 signaling, leading to persistence of the right lateral and regression of the left lateral. Molin et al. examined *Tgfβ2−/−* mice and found significant defects in AA IV, though some mice had normal AA [72]. This study indicated that SMAD2 signaling was critical for the development of AA IV and the authors hypothesized that the WSS levels in the unaffected *Tgfβ2−/−* mice were high enough to maintain Tgfβ1/Alk5 signaling for sufficient SMAD2 levels. More experimental studies are required to link WSS with these molecular mechanisms and our CFD modeling techniques are well suited to determine 3D WSS distributions.

### Modeling 3D blood flow in the embryonic aortic arches

As previously described, our 3D AA models were constructed from a library of micro-CT scans and therefore represent an average embryo. We verify the 3D geometries by comparing AA diameter (Table S1) and length (Table S2), both of which were quantitatively similar. Due to the smaller sample size of experimental length measurements, we further examined the influence of AA length using our previously published numerical parametric 2D hemodynamic model of the right lateral AA [62]. Briefly, this model uses parametrically defined third order Bezier curves to describe the centerlines of the AA and then generates a lumen of uniform diameter by extracting in the normal directions. An outflow tract and dorsal aorta are incorporated at the proximal and distal ends, respectively. We modified this 2D model to represent the 3AA-cranial stage 21 configuration and applied the stage 21 cycle average flow rate to model a steady state simulation (only one half of the total flow was used as we only model the right lateral). As in the 3D models, we employed a rigid wall assumption and 90/10 trunk/cranial flow split at the dorsal aorta. Blood properties remained the same as the 3D models. The parametric geometry allowed us to easily modify AA length and curvature. We performed simulations for 12 distinct geometry cases, varying the lengths and curvatures of each AA individually (Figure S5). We found that when the vessel length varied by 50%, the flow distribution and WSS were maintained within 20% of its original values (Figure S5). Furthermore, when the curvature of the AA was changed such that AA tortuosity increased by 10%, flow distribution and WSS were maintained within 10% of their original values (Figure S5). Therefore, we expect that the small difference between the AA lengths of the 3D models and the experimental measurements (∼3%) does not have a significant effect. While a larger number of experimental length measurements would provide additional evidence, our current data and 2D simulations indicate that even a difference up to 20% in AA length would have little effect on the flow distribution and WSS.

We further qualitatively compared our 3D AA models with previous descriptions of the AA at comparable stages including scanning electron micrographs [8], schematic illustrations [73], reconstructions from serial registered histological sections [74], [75], and MRM [15]. Although 3D information is very limited in these reports, all of these studies are in qualitative agreement with the topology of composite 3D reconstructions in the current study. The integrity of the present 3D quantitative morphology of the AA was further verified 1) by overlapping the 3D reconstructions with the large set of 2D fluorescence dye injection recordings at several views, 2) through multiple snap-shots used for vessel diameter measurements, and 3) by the auxiliary micro-CT scans and 3D reconstructions with parametric segmentation and smoothing settings. These checks were previously applied to our stage 18 and 24 models [39]. It is clear from the longitudinal OCT sections that AA diameter is not constant along the vessel length (Figure 2), and this variation is captured by our 3D models. That the smallest diameter appears at the midpoint is consistent with previous reports that show formations of the AA lumens begin at the aortic sac and dorsal aorta and gradually progress to the midpoint [76], [77]. The strong quantitative and qualitative agreement suggests that our models provide good estimation of the flow and WSS distribution within the embryonic AA at stage 21.

The CFD models used in this study are subject to several assumptions related to boundary conditions. We specify rigid walls, which may over-estimate the WSS values. As we compare these values to our previous stage 18 and 24 models [39], which also employed a rigid wall assumption, the results related to these comparisons remain valid. While the AA wall is distensible, our experience with the chick embryo indicates that the expansion is small during systole (see Movie S1 for a time-lapse OCT sequence). Our rigid wall models provide a good estimation of the biomechanical forces acting on the embryonic AA during this critical stage in development. Measuring flow in these vessels using direct experimental techniques such as Doppler ultrasound or micro particle image velocimetry is difficult and prone to errors given their small size and limited access due to their position within the pharyngeal arches. A full fluid-structure interaction model would be necessary to capture the effects of wall compliance and the surrounding tissue. The outlet boundary conditions specify a 90/10 flow split between the trunk and cranial vessels. As the flow split is enforced by the CFD model, it is independent of the AA morphology. This distribution is based on Doppler ultrasound studies in the chick embryo, and is consistent across the investigated timeframe [52]. The ratio of the cranial and trunk peripheral resistances set this distribution *in vivo*. Though the trunk peripheral resistance decreases geometrically from stages 12 to 29, the ratio likely remains constant since the 90/10 flow split is maintained [52]. Alterations in the peripheral resistance may change this flow split, leading to variations in AA perfusion and therefore WSS. We examined the effects of a 60/40 flow split using our stage 18 model [39] and found that the larger cranial perfusion shifted approximately 5% of the cardiac output from the caudal-most AA pair IV to the cranial-most AA pair II (Table 3). Flow to AA pair III remained similar. Thus, peripheral resistance can have an effect on the AA flow distribution and the biomechanical environment. Indeed, increasing the downstream arterial resistance by ligating the right vitelline artery reduced dorsal aortic flow by 38% after one hour, though cranial flow and AA flow was not measured [31]. Future research is required to determine the effects of altered peripheral resistance on AA flow.

**Table 3:** Stage 18 AA flow distribution in 90/10 and 60/40 trunk/cranial flow split boundary conditions.

	Flow (mm^3^/s)					% Change	
	90/10 split			60/40 split			
AA	R	L		R	L	R	L
II	0.142	0.135		0.149	0.144	4.3	6.4
III	0.265	0.159		0.263	0.156	−0.5	−1.8
IV	0.145	0.055		0.138	0.052	−5.3	−5.4

### Limitations

Although limited published data exists on AA dimensions, our data is consistent with these previous studies [17], [36]. The 2D diameter measurements acquired from fluorescent dye images were influenced by both reflected fluorescent light and the volume of injected dye, requiring the large sample numbers. Due to the large size of pharyngeal arch 2, identifying the boundaries of AA II was sometimes difficult. The imaging depth using OCT is limited by the amount of light scattering caused by the sample and is normally 1.5 mm for biological tissue. At stage 21, pharyngeal arch 2 causes excessive light scattering due to the thickness of the tissue and obstructs imaging of AA II and occasionally AA III when using OCT. Therefore, we did not measure AA II under OCT and did not attempt to classify AA configurations, as errors were likely to result due to the obscured cranial AA. The obstructed imaging of AA III is the reason for the different n-numbers between AA III and IV in Table 1. Measuring AA diameter with OCT assumes a circular cross-section, which we feel is valid. This technique is limited by observer identification of the AA lumen, which is considerably improved when averaging several B-scans.

The four stage 21 AA geometries were created by adding additional AA to the baseline 2AA (III, IV) configuration. We used the same AA vessel geometries in each configuration (i.e. left lateral AA III, 3AA-cranial is the same as left lateral AA III, 3AA-caudal, etc.). This strategy removes any differences in AA diameter or curvature that may exist among the four stage 21 configurations and midpoint AA diameters in the models do not always exactly match those measured experimentally (Table S1). Neglecting these differences when constructing the models may result in an inconsistency between flow rates and WSS levels obtained from CFD and their actual, *in vivo* values. This possible discrepancy may be a reason for deviations in the trend between the change in WSS and change in diameter (Figure 6). A comparison of *in vivo* velocity data at each stage 21 configuration would be necessary to determine the degree of this difference, however no such data currently exists. However, given that AA diameters are similar for all the stage 21 configurations (see above), we expect that our method in creating the 3D model generated little error when comparing flow and WSS values.

## Conclusions

Our study provides the first comparison between quantitative *in vivo* data and CFD-predicted flow and WSS patterns of the stage 21 embryonic AA. We have shown a transient variability in the number and identity of AA present at stage 21, creating four possible configurations. We applied multimodal imaging strategies to provide the first quantitative data on AA diameter at stage 21, which revealed significant growth in key AA vessels (IV, VI) when compared to our previous data at stage 18, and asymmetric growth of AA IV when compared to our stage 24 data (right lateral grew significantly, left lateral remained the same). CFD analysis of all four stage 21 configurations demonstrated changes in cardiac output distribution and elevated WSS levels compared to stages 18 and 24. Our data revealed that changes in WSS and AA diameter are closely correlated, providing further evidence for flow-dependency in embryonic vascular growth. In particular, flow asymmetry in AA IV may relate to its asymmetric growth patterns based on shear-mediated gene expression and signaling activation. The timing of events such as cardiac output increase and outflow tract migration may have additional roles in the progression of AA growth and remodeling. Understanding the relationship between hemodynamics and the growth of the AA can provide insight into the progression of great vessel defects and other forms of CHD.

## Supporting Information

Figure S1 **Diameter measurement of a nylon filament using OCT.** Each panel (A–D) represents the diameter computed based on the selected points (green dots). The best fit circle is shown in yellow and the diameter is given at the center. The distribution of the selected points around the circumference of the fiber did not significantly affect the calculated diameter. This method is sufficient to measure AA diameters from transverse sections where the entire lumen boundary is not visible.
(TIF)

Figure S2 **The pulsatile flow waveform used to represent a single cardiac cycle at the outflow tract for the CFD model was interpolated from the data published by Yoshigi et al.** [**51**] **.**
(TIF)

Figure S3 **Graphical comparison of average AA midpoint diameter (±SD), cardiac cycle-averaged flow, and spatially-averaged (±SD) cycle-average WSS levels for each of the four configurations at stage 21 with the preceding (stage 18) and succeeding (stage 24) data from our previous work** [**39**] **.** Widths of bars are scaled, with values provided for stage 21. Gray boundaries give the SD. The rate of change of diameter, flow, and WSS is dependent on the stage 21 AA configuration. Significant differences (p<0.05) between stage 21 diameters are designated with *, where superscripts delineate the statistical pairs.
(TIF)

Figure S4 **AA WSS distributions at peak flow in the 3AA-cranial configuration with a parabolic inlet profile (A), and skewed parabolic inlet profile (B).** The velocity cross section shows the profile shape at the outflow tract. Compare to Figure 5A.
(TIF)

Figure S5 **Effect of AA length and curvature on flow distribution and WSS.** Our parametric 2D CFD model of the 3AA-cranial stage 21 configuration was used to simulate 12 distinct AA geometries, varying the lengths and curvatures of each individual AA. The velocity fields for each case, numbered 1–12, are depicted. The base model (case 0) is shown in the lower left. Panels A–E show the variation in flow rate (A), WSS (B), resistance (C), length (D), and tortuosity (E) compared to the base model. Resistance is computed as the pressure drop from the outflow tract to the outlet of the AA, divided by the flow through the AA. Tortuosity is the length of the AA divided by the Euclidean distance between its endpoints. Q – flow rate, R – resistance, L – length, T – tortuosity.
(TIF)

Table S1 **Experimentally measured average (±SD) AA diameters compared with those in the 3D models used for CFD simulations.** See Table 1 for experimental sample sizes.
(DOC)

Table S2 **Experimentally measured AA lengths compared with those in the 3D models used for CFD simulations.** Experimental measurements were taken for a single AA sample (n = 1) and one measurement was made per sample.
(DOC)

Table S3 **Average stage 21 AA diameter (±SD) data compared with our previous stage 18 and stage 24 data**.
(DOC)

Movie S1 **Time-lapse OCT sequence through a transverse section of the right lateral AA at stage 21.** The movie is obtained *in vivo* with no embryonic intervention and during the regular cardiac cycle of the beating ventricle. Sections of AA III and IV are visible, where AA IV is towards the left. AA II can also be seen furthest to the right, within the large pharyngeal arch 2. Cranial is toward the right and dorsal is into the page (same as Figure 2C). Frame dimensions are 1.5×1.5 mm.
(AVI)
