# Supplementary figures and images for: Critical Transitions in Early Embryonic Aortic Arch Patterning and Hemodynamics
Source: PLoS One. 2013 Mar 21;8(3):e60271. doi: 10.1371/journal.pone.0060271 (PMC3605337; doi:10.1371/journal.pone.0060271)

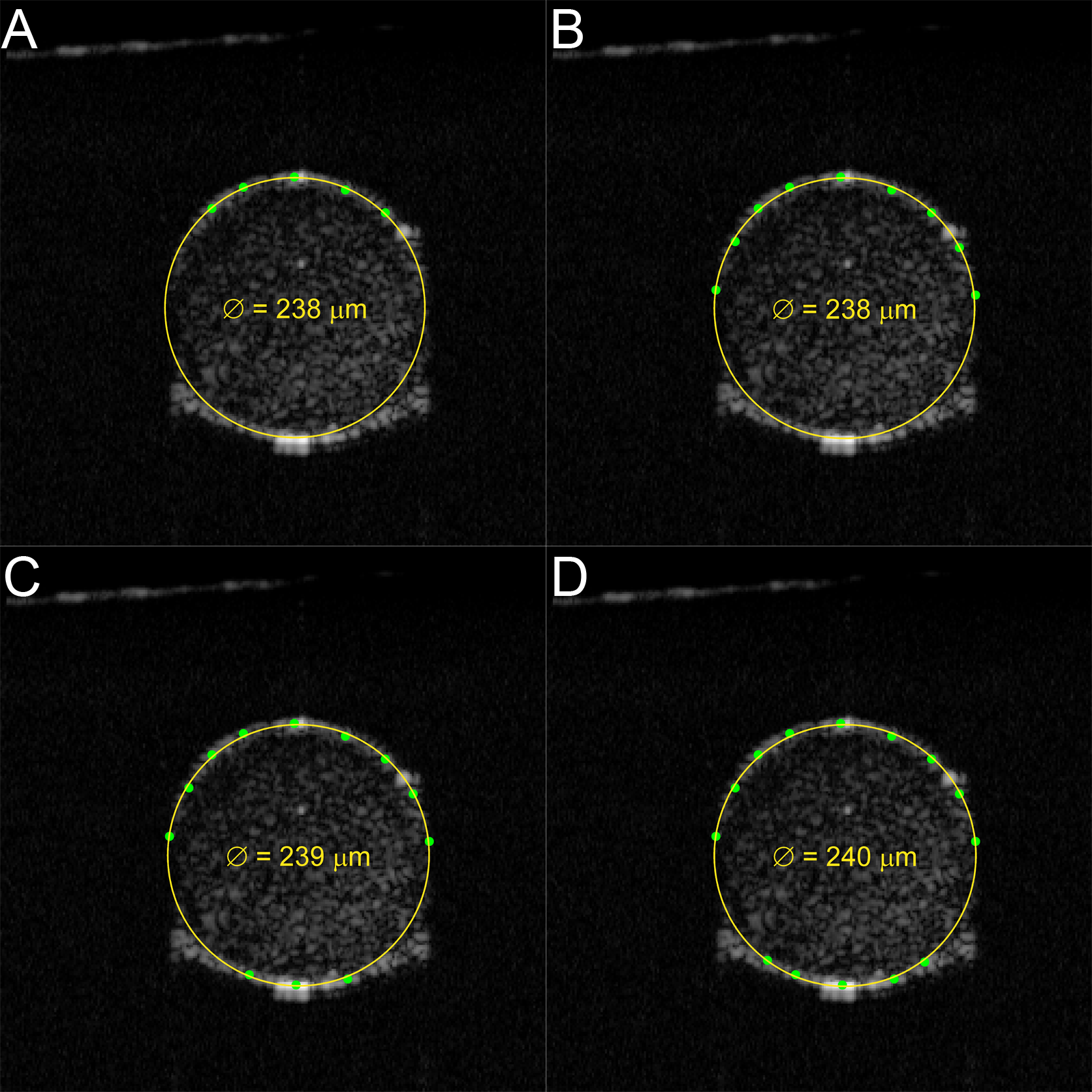

Supplement: Figure S1 — Diameter measurement of a nylon filament using OCT. Each panel (A–D) represents the diameter computed based on the selected points (green dots). The best fit circle is shown in yellow and the diameter is given at the center. The distribution of the selected points around the circumference of the fiber did not significantly affect the calculated diameter. This method is sufficient to measure AA diameters from transverse sections where the entire lumen boundary is not visible. (TIF) [file pone.0060271.s001.tif]

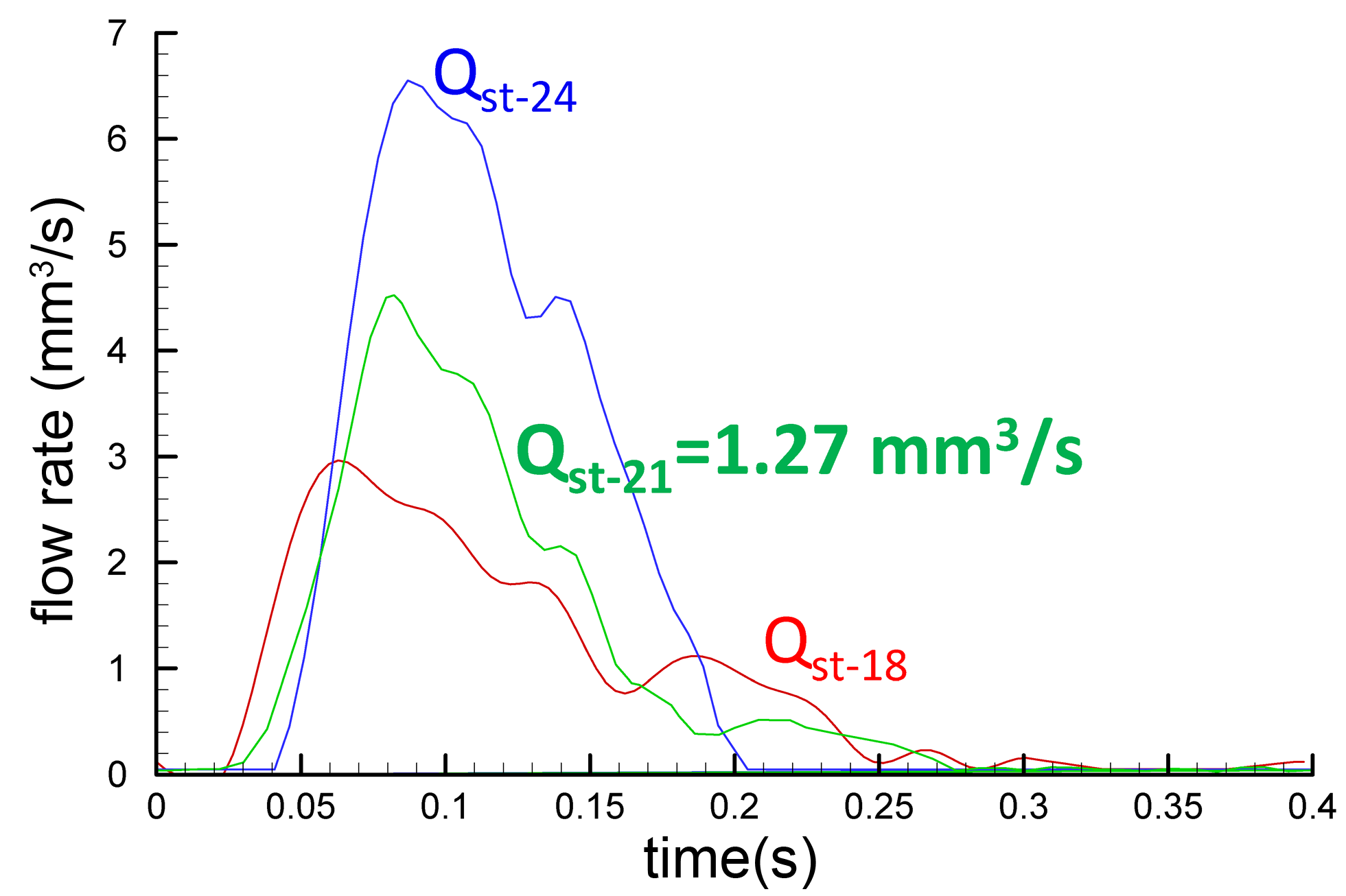

Supplement: Figure S2 — The pulsatile flow waveform used to represent a single cardiac cycle at the outflow tract for the CFD model was interpolated from the data published by Yoshigi et al. [51] . (TIF) [file pone.0060271.s002.tif]

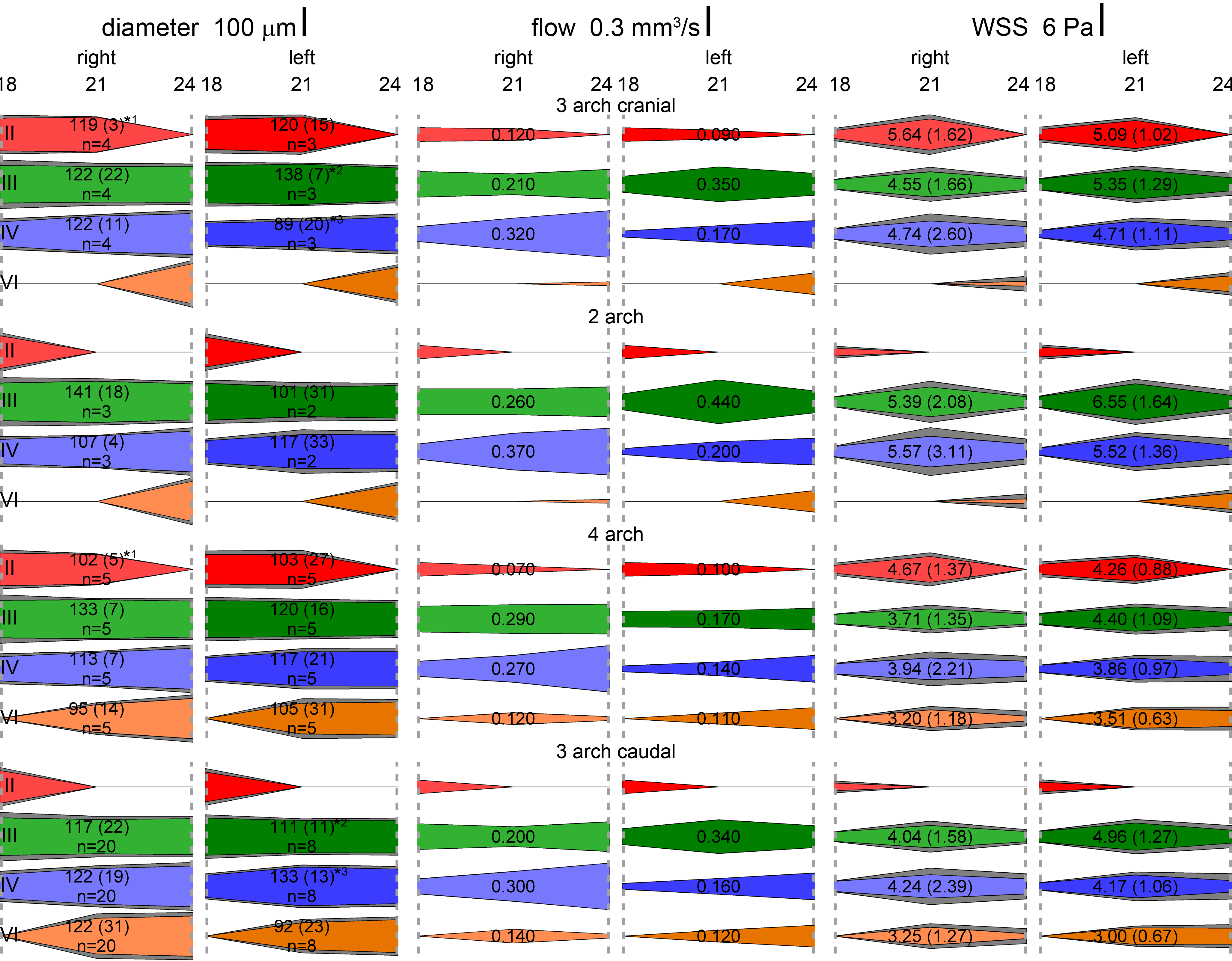

Supplement: Figure S3 — Graphical comparison of average AA midpoint diameter (±SD), cardiac cycle-averaged flow, and spatially-averaged (±SD) cycle-average WSS levels for each of the four configurations at stage 21 with the preceding (stage 18) and succeeding (stage 24) data from our previous work [39] . Widths of bars are scaled, with values provided for stage 21. Gray boundaries give the SD. The rate of change of diameter, flow, and WSS is dependent on the stage 21 AA configuration. Significant differences (p<0.05) between stage 21 diameters are designated with *, where superscripts delineate the statistical pairs. (TIF) [file pone.0060271.s003.tif]

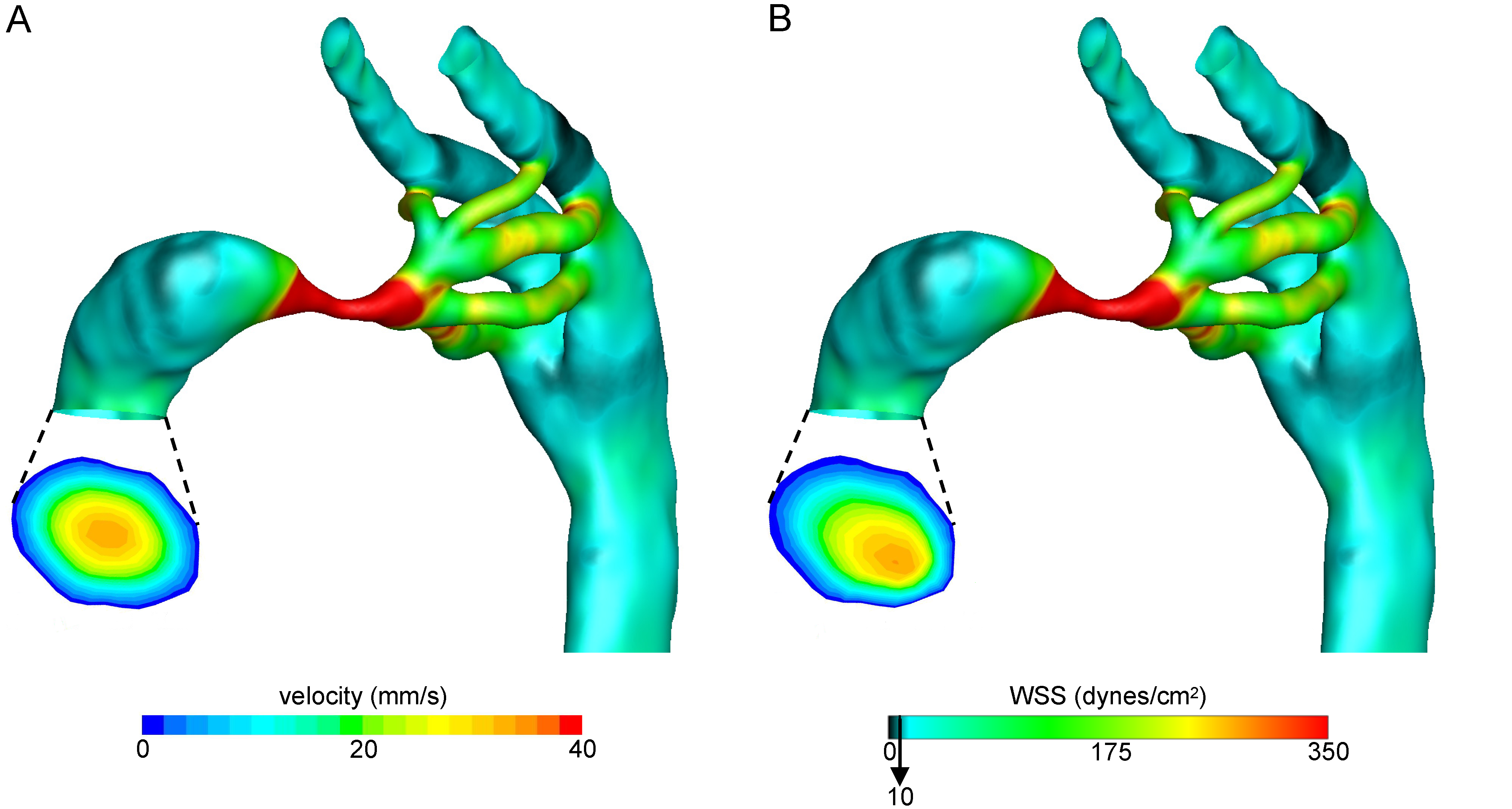

Supplement: Figure S4 — AA WSS distributions at peak flow in the 3AA-cranial configuration with a parabolic inlet profile (A), and skewed parabolic inlet profile (B). The velocity cross section shows the profile shape at the outflow tract. Compare to Figure 5A. (TIF) [file pone.0060271.s004.tif]

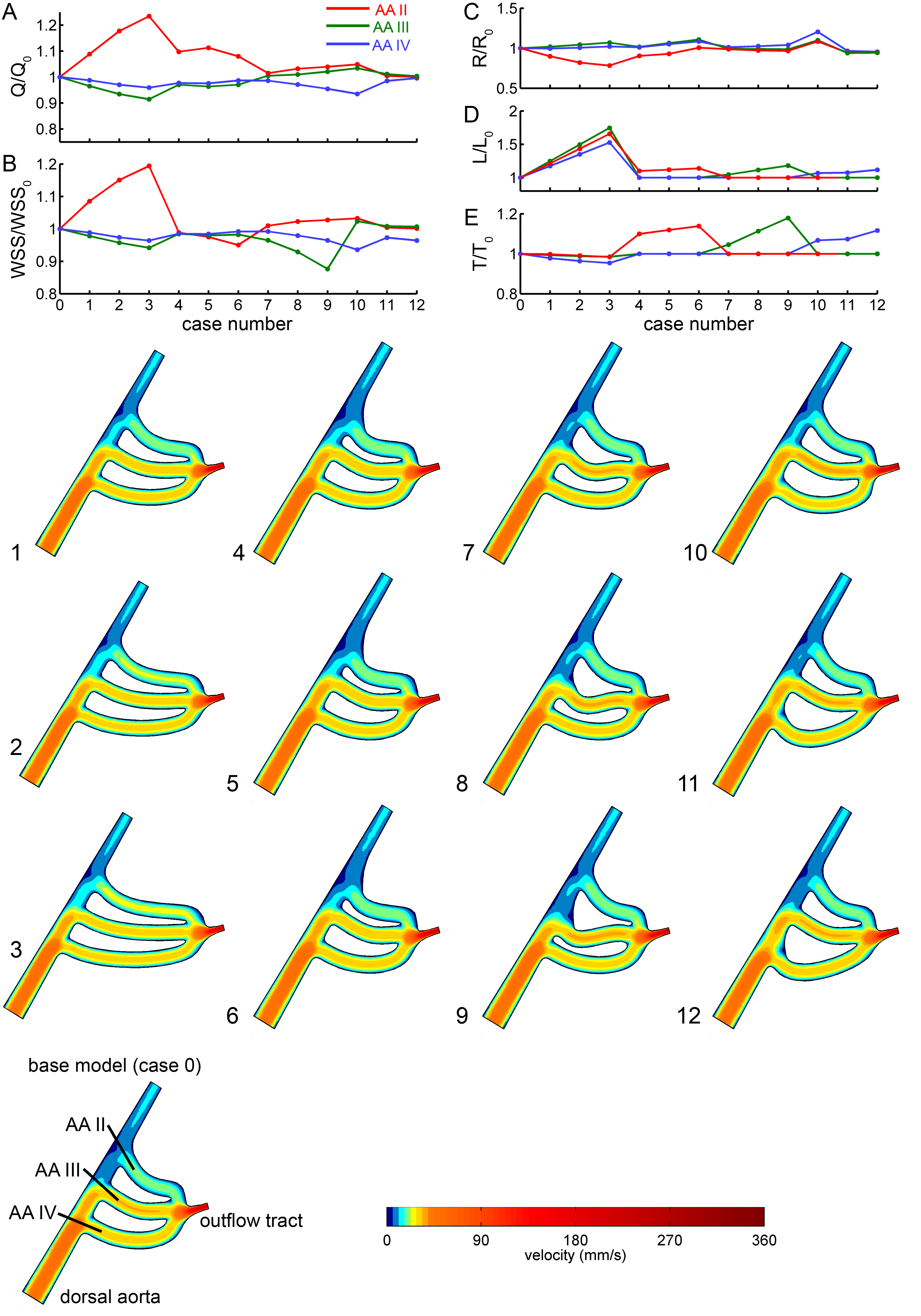

Supplement: Figure S5 — Effect of AA length and curvature on flow distribution and WSS. Our parametric 2D CFD model of the 3AA-cranial stage 21 configuration was used to simulate 12 distinct AA geometries, varying the lengths and curvatures of each individual AA. The velocity fields for each case, numbered 1–12, are depicted. The base model (case 0) is shown in the lower left. Panels A–E show the variation in flow rate (A), WSS (B), resistance (C), length (D), and tortuosity (E) compared to the base model. Resistance is computed as the pressure drop from the outflow tract to the outlet of the AA, divided by the flow through the AA. Tortuosity is the length of the AA divided by the Euclidean distance between its endpoints. Q – flow rate, R – resistance, L – length, T – tortuosity. (TIF) [file pone.0060271.s005.tif]
