# Supplementary material for: Critical Transitions in Early Embryonic Aortic Arch Patterning and Hemodynamics
Source: PLoS One. 2013 Mar 21;8(3):e60271. doi: 10.1371/journal.pone.0060271 (PMC3605337; doi:10.1371/journal.pone.0060271)
Supplement: Table S1 — Experimentally measured average (±SD) AA diameters compared with those in the 3D models used for CFD simulations. See Table 1 for experimental sample sizes. (DOC) [file pone.0060271.s006.doc]

**Table S1.** Experimentally measured average (±SD) AA diameters compared with those in the 3D models used for CFD simulations. See Table 1 for experimental sample sizes.

| AA | lateral | Measured diameter (±SD) (mm) | | Model diameter (mm) |
| --- | --- | --- | --- | --- |
| II | R | 0.109 | (0.009) | 0.092 |
|  | L | 0.110 | (0.024) | 0.086 |
| III | R | 0.125 | (0.020) | 0.123 |
|  | L | 0.114 | (0.019) | 0.145 |
| IV | R | 0.123 | (0.021) | 0.148 |
|  | L | 0.115 | (0.021) | 0.114 |
| VI | R | 0.118 | (0.026) | 0.122 |
|  | L | 0.094 | (0.027) | 0.123 |
