# Supplementary material for: Critical Transitions in Early Embryonic Aortic Arch Patterning and Hemodynamics
Source: PLoS One. 2013 Mar 21;8(3):e60271. doi: 10.1371/journal.pone.0060271 (PMC3605337; doi:10.1371/journal.pone.0060271)
Supplement: Table S2 — Experimentally measured AA lengths compared with those in the 3D models used for CFD simulations. Experimental measurements were taken for a single AA sample (n = 1) and one measurement was made per sample. (DOC) [file pone.0060271.s007.doc]

**Table S2.** Experimentally measured AA lengths compared with those in the 3D models used for CFD simulations. Experimental measurements were taken for a single AA sample (n=1) and one measurement was made per sample.

| AA | lateral | Measured length (mm) | Model length (mm) | % difference |
| --- | --- | --- | --- | --- |
| II | R |  | 0.408 |  |
|  | L |  | 0.412 |  |
| III | R |  | 0.736 |  |
|  | L | 0.610 | 0.615 | 0.89% |
| IV | R | 0.706 | 0.740 | 4.78% |
|  | L | 0.655 | 0.725 | 10.71% |
| VI | R | 0.613 | 0.596 | -2.81% |
|  | L |  | 0.405 |  |
| average | | 0.646 | 0.580 | 3.40% |
