# Supplementary material for: Critical Transitions in Early Embryonic Aortic Arch Patterning and Hemodynamics
Source: PLoS One. 2013 Mar 21;8(3):e60271. doi: 10.1371/journal.pone.0060271 (PMC3605337; doi:10.1371/journal.pone.0060271)
Supplement: Table S3 — Average stage 21 AA diameter (±SD) data compared with our previous stage 18 and stage 24 data. (DOC) [file pone.0060271.s008.doc]

**Table S3.** Average stage 21 AA diameter (±SD) data compared with our previous stage 18 and stage 24 data^1^.

|  |  | Midpoint diameter (±SD) (mm) | | | *n* (number of embryos) | | |
| --- | --- | --- | --- | --- | --- | --- | --- |
| AA | lateral | stage 18 | stage 21 | stage 24 | stage 18 | stage 21 | stage 24 |
| II | R | 0.112 | 0.109 |  | 23 | 9 |  |
|  |  | (0.021) | (0.009) |  |  |  |  |
|  | L | 0.101 | 0.110 |  | 33 | 8 |  |
|  |  | (0.025) | (0.024) |  |  |  |  |
| III | R | 0.129 | 0.125 | 0.123 | 23 | 39 | 30 |
|  |  | (0.037) | (0.020) | (0.016) |  |  |  |
|  | L | 0.127 | 0.114 | 0.112 | 33 | 27 | 24 |
|  |  | (0.023) | (0.019) | (0.015) |  |  |  |
| IV | R | 0.083^*^ | 0.123^*,‡^ | 0.140^‡^ | 23 | 49 | 30 |
|  |  | (0.020) | (0.021) | (0.024) |  |  |  |
|  | L | 0.075^†^ | 0.115^†^ | 0.118 | 29 | 28 | 24 |
|  |  | (0.011) | (0.021) | (0.019) |  |  |  |
| VI | R |  | 0.118^♦^ | 0.138^♦^ |  | 41 | 29 |
|  |  |  | (0.026) | (0.024) |  |  |  |
|  | L |  | 0.094^♣^ | 0.113^♣^ |  | 14 | 24 |
|  |  |  | (0.027) | (0.019) |  |  |  |

*,†,‡,♦,♣ indicate a statistically significant difference (p<0.05) between AA diameters.

^1^Wang Y, Dur O, Patrick MJ, Tinney JP, Tobita K, et al. (2009) Aortic arch morphogenesis and flow modeling in the chick embryo. Annals of Biomedical Engineering 37: 1069-1081.
